# Association Between TERT rs2736098 Polymorphisms and Cancer Risk-A Meta-Analysis

**DOI:** 10.3389/fphys.2018.00377

**Published:** 2018-04-11

**Authors:** Mi Zhou, Bo Jiang, Mao Xiong, Xin Zhu

**Affiliations:** ^1^Department of Respiratory Medicine, The First Affiliated Hospital of Chongqing Medical University, Chongqing, China; ^2^Department of Urology, The First Affiliated Hospital of Chongqing Medical University, Chongqing, China

**Keywords:** TERT, single nucleotide polymorphism, cancer, meta-analysis, genetic

## Abstract

**Background:** Cancer remains a leading cause of death and constitutes an enormous burden on society worldwide. The association between the human telomerase reverse transcriptase (TERT) gene variant rs2736098 polymorphisms and cancer predisposition remain inconclusive.

**Objective and methods:** Databases including Pubmed and Embase were systematically searched from inception to September 15, 2017 to retrieve studies investigating the association between the TERT variant rs2736098 polymorphisms and cancer risk in accordance with previously determined exclusion and inclusion criteria. The pooled odds ratios (ORs) and corresponding 95% confidence intervals (CIs) were evaluated using random or fixed effects models.

**Results:** Thirty-one case-control studies from 29 articles with 15,837 cases and 19,263 controls were screened out after a systematic search. Pooled analysis demonstrated that the TERT variant rs2736098 G > A polymorphism was significantly correlated with cancer risk in all populations (A vs. G: OR = 1.134, 95% CI = 1.051–1.224, *P* = 0.001; AA vs. GG: OR = 1.280, 95% CI = 1.087–1.508, *P* = 0.003; GA vs. GG: OR = 1.125, 95% CI = 1.020–1.240, *P* = 0.018; GA/AA vs. GG: OR = 1.159, 95% CI = 1.047–1.283, *P* = 0.004). In the subgroup analysis based on cancer type, the TERT rs2736098 with the A allele was 1.299 times more frequent than that with the G allele (OR = 1.299, 95% CI = 1.216–1.386) under the allelic genetic model in lung cancer, and 1.152 times (OR = 1.152, 95% CI = 1.032–1.286) that in bladder cancer.

**Conclusions:** This meta-analysis demonstrated significant correlations between the TERT variant rs2736098 polymorphisms and cancer susceptibility. The A allele in the rs2736098 G > A polymorphism contributes to susceptibility in many types of cancer, especially lung cancer and bladder cancer.

## Introduction

Cancer remains a leading cause of death and constitutes an enormous burden on society in both developed and developing countries, with an estimated 14.1 million newly diagnosed patients and high cancer-related mortality worldwide in 2012 (Torre et al., 2015). The occurrence and development of cancer are associated with a complex interaction between genetic and environmental factors. Genome-wide association (GWA) studies have identified common sequence variants that affect cancer risk (Gudmundsson et al., 2007), and sequence variants at the telomerase reverse transcriptase (TERT)- cleft lip and palate transmembrane 1-like(CLPTM1L) locus of the human 5p15.33 chromosome are connected with various types of cancer, such as lung cancer, prostate cancer, and bladder cancer (Rafnar et al., 2009).

TERT, located in the human 5p15.33 locus, encodes a catalytic subunit of telomerase and exerts a pivotal role in the maintenance of telomere DNA length and carcinogenesis (Rafnar et al., 2009). Many studies have assessed the relationship between TERT gene polymorphisms and cancer susceptibility; however, studies have yet to reach a consensus. Xiao et al. found that the risk of developing lung cancer with the TERT rs2736098 polymorphism carrying the A allele was 1.343 times higher than that associated with the G allele in Chinese males (Xiao and He, 2017). In renal cell cancer, the AA genotype of TERT rs2736098 is associated with increased cancer susceptibility and decreased telomere length compared with the GG genotype (de Martino et al., 2016). However, in breast cancer, the A allele of TERT rs2736098 shows a protective effect against breast cancer susceptibility in the Iranian population (Hashemi et al., 2014; Oztas et al., 2016).

Previous studies reached paradoxical conclusions regarding the association between TERT rs2736098 polymorphisms and cancer risk. Since new studies were recently published and certain ignored studies were retrieved, we performed a meta-analysis to comprehensively assess the correlation between rs2736098 polymorphisms and cancer susceptibility. In addition, ethnicity-specific and cancer-specific effects on the correlation were evaluated through subgroup analysis.

## Materials and methods

### Publication search and inclusion criteria

A systematic search of databases including PubMed and Embase was conducted to retrieve studies investigating the association between rs2736098 polymorphisms and various types of cancer, published up to September 15, 2017. The search terms were as follows: “TERT,” “telomerase reverse transcriptase,” “5p15,” “TERT-CLPTM1L,” “polymorphism,” “polymorphisms,” “cancer,” “tumor,” “neoplasm,” and “carcinoma.” Relevant journals and the references of the included studies were screened as well. The inclusion criteria for the meta-analysis were as follows: (1) a case-control design; (2) investigating TERT rs2736098 polymorphisms and cancer susceptibility; and (3) detailed numbers of alleles and genotypes between cases and controls. The exclusion criteria were (1) reviews or case reports without control; (2) no available data presented; and (3) duplication of reports.

### Data extraction

Two investigators (Zhou and Jiang) screened the literature and extracted data independently in strict accordance with the above exclusion and inclusion criteria. Discrepancies and differences were presented to a third investigator (Zhu) until consensus was reached. The collected information was as follows: first author' name, publication year, country, ethnicity, sample size of case and control groups, genotyping methods, allele genotypes of each gene polymorphism, and *P*-value for Hardy-Weinberg Equilibrium (HWE).

### Statistical analyses

The odds ratios (ORs) and their corresponding 95% confidence intervals (CIs) were applied to evaluate the strength of the association between polymorphisms in rs2736098 and cancer susceptibility. Five genetic models were used to calculate pooled ORs, including the dominant (GA + AA vs. GG), recessive (AA vs. GA + GG), heterozygous (GA vs. GG), homozygous (AA vs. GG), and allelic (A vs. G) models. The Chi square-based Q-test was used to assess between-study heterogeneity and a *P*-value of 0.1 was interpreted as significant heterogeneity among studies (Lau et al., 1997). The I2 metric value was also used to describe the proportion of total variation in study estimates that is due to heterogeneity with values of 25, 50, and 75% considered as evidence of low, moderate, and high heterogeneity, respectively (Higgins and Thompson, 2002). The pooled OR estimation of each study was calculated by the fixed-effects model (the Mantel–Haenszel method) when *P* was > 0.10 and I2 was < 50% (Mantel and Haenszel, 1959). Otherwise, the random-effects model (the DerSimonian and Laird method) was used (DerSimonian and Laird, 1986). Subgroup analyses according to ethnicity were performed to calculate the ethnic-specific OR. HWE was tested for each study by comparing the observed and expected genotype frequencies of the control group (Chi-square test). Publication bias was evaluated with Egger's linear regression and Begg's funnel plots (Hayashino et al., 2005). Statistical analyses were performed using Stata software (version 14.1; Stata Corp, College Station, TX, USA) with a two-sided *P*-value. *P* < 0.05 was considered as significant.

## Results

### Studies and populations

**Figure 1:**
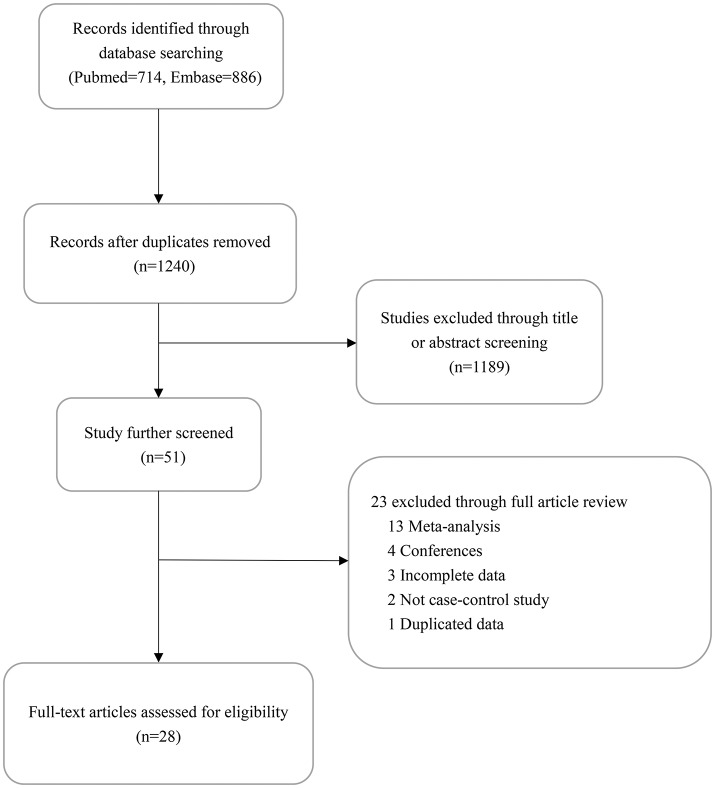
Flow Diagram describing literature selection.

The flow process for retrieval and selection of included studies is shown in Figure 1. After removing duplicates, 1,240 articles were identified through the initial retrieval process. Of these, 1,189 articles were initially excluded through title or abstract screening. Among the remaining 51 articles, 13 were 13 were meta-analyses, four were conferences, three reported incomplete data, two had no controls, and one had duplicated data. Finally, 15,837 cases and 19,263 controls of 31 studies in 28 articles were included in this meta-analysis (Savage et al., 2007; Choi et al., 2009; Liu et al., 2010; Chen et al., 2011; Ding et al., 2011; Gago-Dominguez et al., 2011; Jaworowska et al., 2011; Hofer et al., 2012; Wang et al., 2012, 2016; Li et al., 2013; Ma et al., 2013; Wu et al., 2013; Zhang et al., 2013, 2014; Gao et al., 2014; Hashemi et al., 2014; Singh et al., 2014; Su et al., 2014; Yin et al., 2014; Zhao et al., 2014; Jannuzzi et al., 2015; Carkic et al., 2016; de Martino et al., 2016; Oztas et al., 2016; Xing et al., 2016; Xiao and He, 2017; Yuan et al., 2017). The genotyping methods included TaqMan PCR, PCR-RFLP, Sequenom, and LDR. The genotype distribution of the controls in all studies was consistent with HWE except in three studies (Hofer et al., 2012; Jannuzzi et al., 2015; Carkic et al., 2016; Table 1).

**Table 1:** Summary of meta-analysis of association between the TERT variant rs2736098 polymorphisms and cancer risk.

**Variables**	***N***	**A vs. G**			**AA vs.GG**			**GA vs.GG**			**GA/AA vs.GG**			**AA vs.GA/GG**		
		**OR(95%Cl)**	***P*_h_**	***I*^2^(%)**	**OR(95%Cl)**	***P*_h_**	***I*^2^(%)**	**OR(95%Cl)**	***P*_h_**	***I*^2^(%)**	**OR(95%Cl)**	***P*_h_**	***I*^2^(%)**	**OR(95%Cl)**	***P*_h_**	***I*^2^(%)**
All	31	1.134(1.051–1.224)	0	79.3	1.280(1.087–1.508)	0	76.9	1.125(1.020-1.240)	0	73.6	1.159(1.047–1.283)	0	78.3	1.223(1.082–1.384)	0	64.6
**CANCER TYPE**																
Lung Cancer	10	1.299(1.216–1.386)	0.285	17.1	1.796(1.575–2.047)	0.907	0	1.199(1.086–1.323)	0.211	25.2	1.305(1.188–1.434)	0.197	26.8	1.653(1.461–1.869)	0.97	0
Bladder Cancer	6	1.152(1.032–1.286)	0.079	49.3	1.345(1.058–1.708)	0.096	46.5	1.097(0.940–1.279)	0.092	47.2	1.149(0.990–1.334)	0.077	49.8	1.297(1.062–1.586)	0.178	34.4
Breast Cancer	3	0.816(0.670–0.994)	0.107	55.2	0.639(0.458–0.892)	0.235	30.9	0.872(0.675–1.127)	0.194	39	0.796(0.592–1.070)	0.117	53.3	0.707(0.566–0.884)	0.361	1.9
Hepatocellular Carcinoma	3	1.211(0.948–1.548)	0.057	65.1	1.179(0.711–1.956)	0.081	60.2	1.595(1.246–2.042)	0.254	27.1	1.494(1.123–1.987)	0.133	50.4	0.921(0.617–1.376)	0.161	45.3
Colorectal Cancer	2	0.717(0.562–0.915)	0.371	0	0.477(0.276–0.825)	0.611	0	0.539(0.207–1.401)	0.047	74.6	0.592(0.312–1.121)	0.097	63.8	0.726(0.462–1.141)	0.63	0
Renal Cell Carcinoma	1	2.081(1.642–2.637)	–	–	4.934(2.922–8.332)	−	−	4.107(2.495–6.761)	−	−	4.424(2.752–7.112)	–	–	1.811(1.275–2.571)	–	–
Oral squamous cell carcinoma	1	0.518(0.341–0.786)	–	–	0.230(0.076–0.697)	–	–	0.243(0.120–0.492)	–	–	0.241(0.121–0.482)	–	–	0.618(0.232–1.647)	–	–
Esophageal Cancer	1	1.047(0.889–1.234)	–	–	1.146(0.799–1.645)	–	–	0.998(0.787–1.265)	–	–	1.027(0.820–1.286)	–	–	1.148(0.818–1.610)	–	–
Cervic Cancer	1	1.118(0.984–1.269)	–	–	1.335(1.025–1.739)	–	–	0.979(0.809–1.186)	–	–	1.059(0.884–1.267)	–	–	1.350(1.059–1.721)	–	–
Glioma	1	1.194(1.050–1.359)	–	–	1.476(1.113–1.959)	–	–	1.162(0.961–1.405)	–	–	1.223(1.021–1.465)	–	–	1.359(1.045–1.768)	–	–
Laryngeal Cancer	1	1.066(0.842–1.351)	–	–	0.750(0/378–1.490)		–	1.239(0.923–1.664)	–	–	1.170(0.881–1.554)	–	–	0.694(0.353–1.367)	–	–
SCCHN	1	0.923(0.807–1.055)	–	–	0.904(0.643–1.271)	–	–	0.890(0.747–1.061)	–	–	0.892(0.755–1.055)	–	–	0.951(0.682–1.325)	–	–
**ETHNICITY**																
Asian	20	1.217(1.138–1.301)	0	59.7	1.509(1.327–1.716)	0.009	48.3	1.170(1.066–1.284)	0.001	56	1.240(1.132–1.358)	0	59	1.396(1.245–1.565)	0.02	43.6
Caucasian	11	0.965(0.818–1.140)	0	85.9	0.875(0.588–1.302)	0	85	0.984(0.785–1.235)	0	84.7	0.964(0.765–1.214)	0	87	0.928(0.730-1.181)	0.001	65.5

*CI, confidence interval, OR, odds ratio, I^2^, the variation in OR attributable to heterogeneity SCCHN, squamous cell carcinoma of the head and neck*.

### Pooled analyses

Among all the populations, the rs2736098 (G > A) polymorphism was significantly associated with increased cancer risk under the allelic A vs. G genetic model (OR = 1.134, 95% CI = 1.051–1.224, *P* = 0.001), homozygous AA vs. GG genetic model (OR = 1.280, 95% CI = 1.087–1.508, *P* = 0.003), heterozygous GA vs. GG genetic model (OR = 1.125, 95% CI = 1.020–1.240, *P* = 0.018), dominant GA/AA vs. GG genetic model (OR = 1.159, 95% CI = 1.047–1.283, *P* = 0.004), and recessive AA vs. GA/GG genetic model (OR = 1.223, 95% CI = 1.082–1.384, *P* = 0.001) (Table 1, Supplementary Figures 1–5).

Subgroup analysis based on ethnicity detected no obvious association between rs2736098 (G>A) polymorphism and susceptibility to cancer in Caucasian population under five genetic models. However, in the Asian population, the rs2736098 (G>A) polymorphism was significantly associated with cancer risk under allelic A vs. G genetic model (OR = 1.217, 95% CI = 1.138–1.301, *P* = 0.000), homozygous AA vs. GG genetic model (OR = 1.509, 95% CI = 1.327–1.716, *P* = 0.000), heterozygous GA vs. GG genetic model (OR = 1.170, 95% CI = 1.066–1.284, *P* = 0.001); dominant GA/AA vs. GG genetic model (OR = 1.240, 95% CI = 1.132–1.358, *P* = 0.000), and recessive AA vs. GA/GG genetic model(OR = 1.396, 95% CI = 1.245–1.565,*P* = 0.000) (Table 1, Supplementary Figures 1–5).

**Figure 2:**
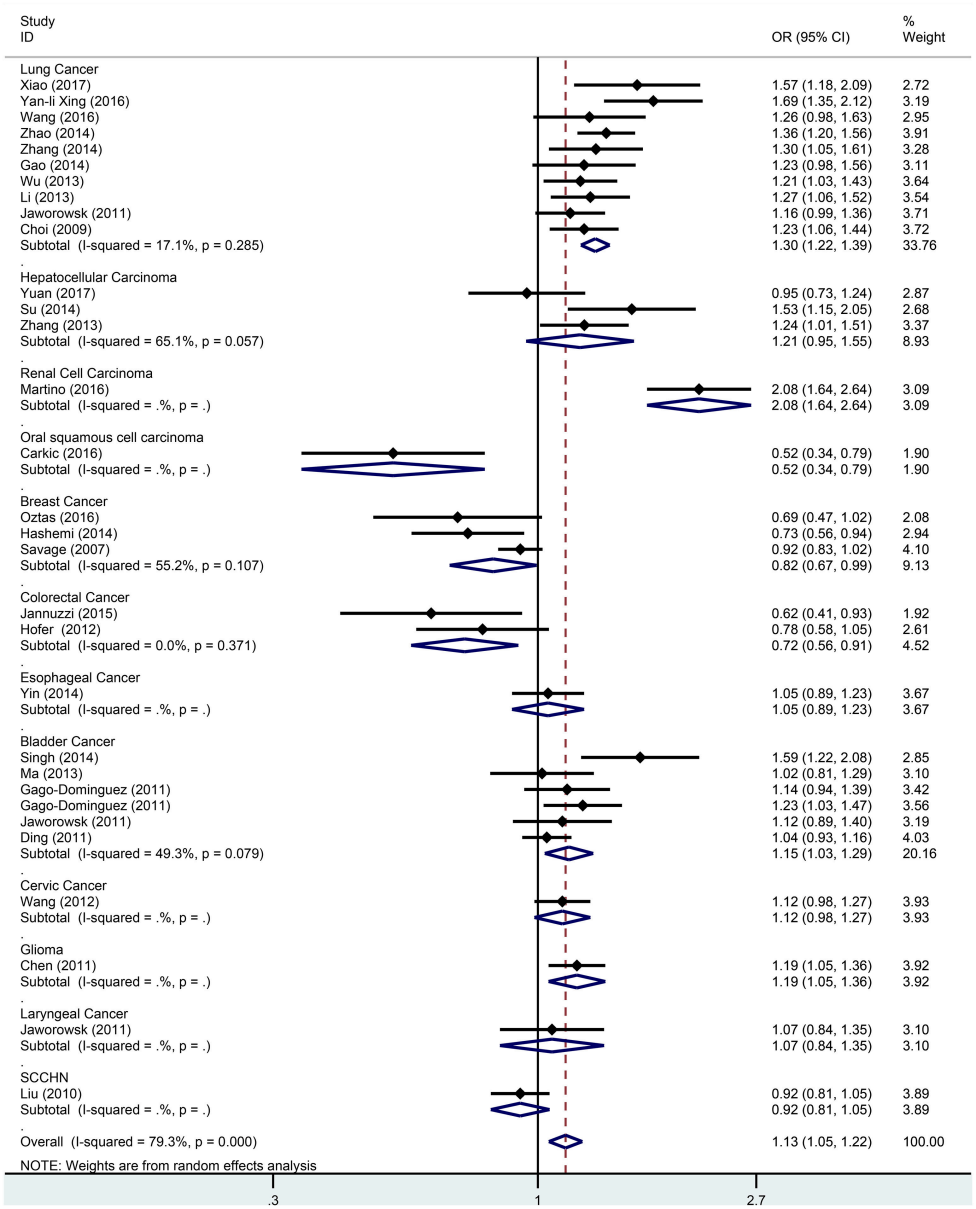
Forest plot for association between the TERT variant rs2736098 polymorphisms and cancer risk under a allele genetic model (A vs. G) after stratification analysis by cancer.

**Figure 3:**
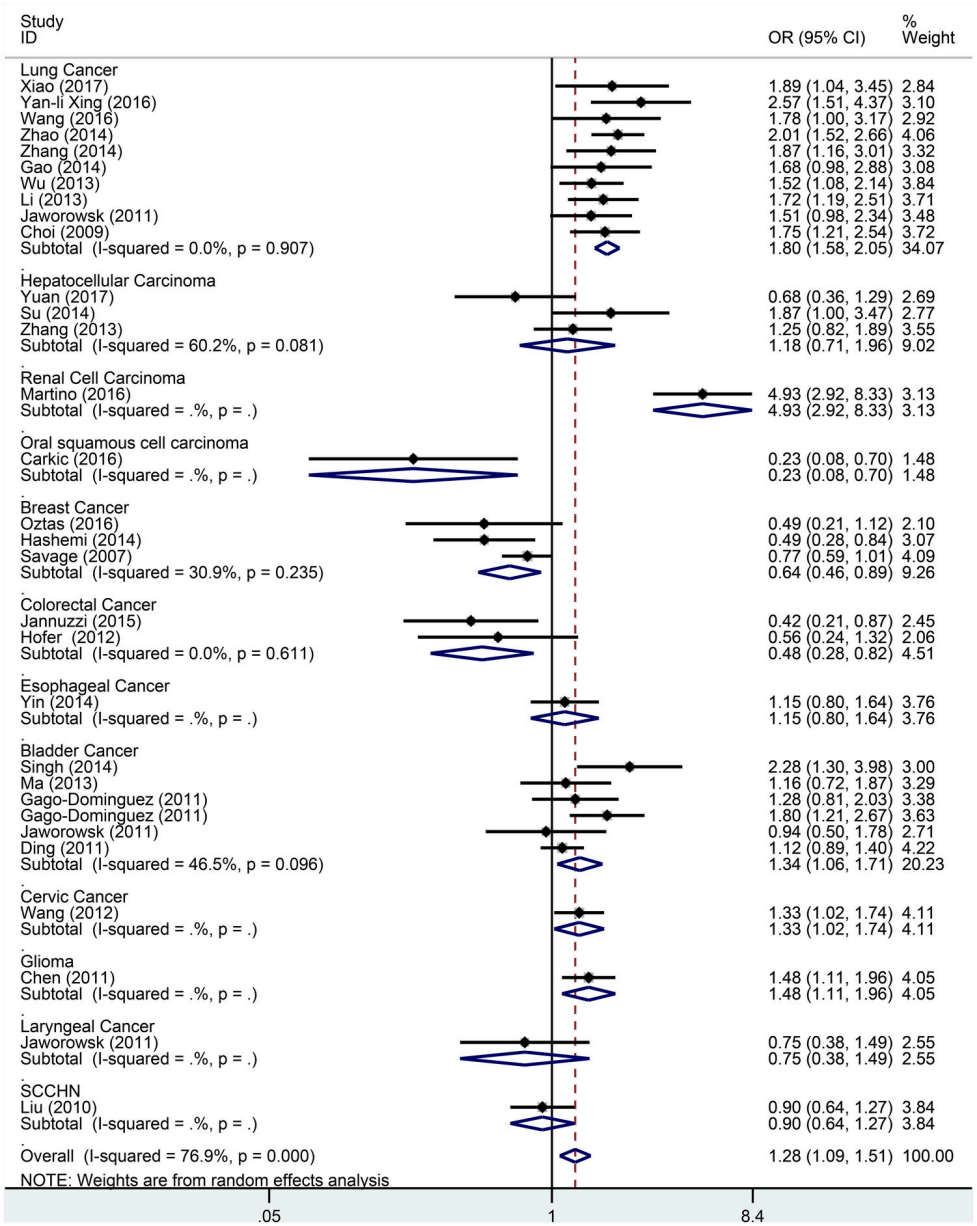
Forest plot for association between the TERT variant rs2736098 polymorphisms and cancer risk under a homozygote genetic model (AA vs. GG) after stratification analysis by cancer.

**Figure 4:**
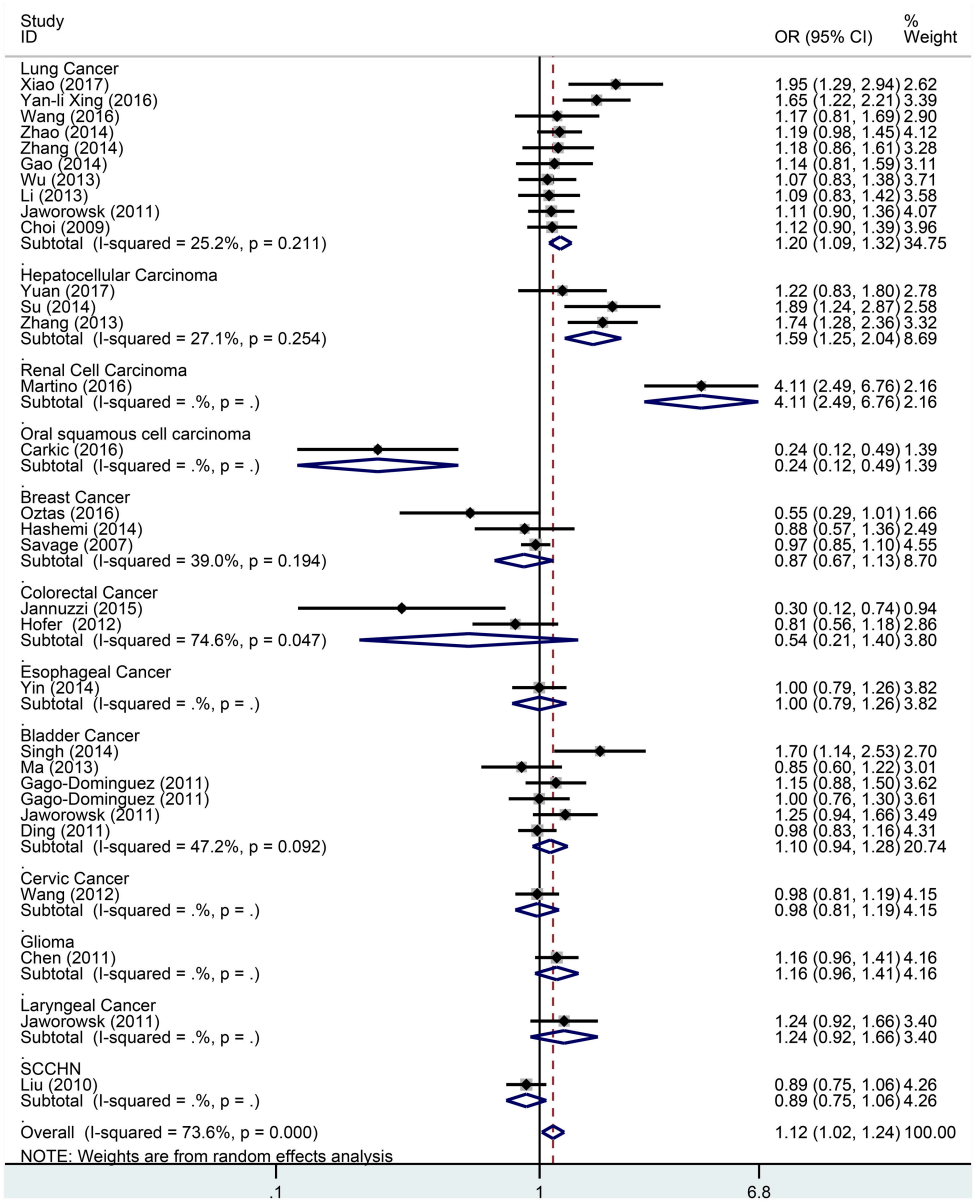
Forest plot for association between the TERT variant rs2736098 polymorphisms and cancer risk under a heterozygote genetic model (GA vs. GG) after stratification analysis by cancer.

**Figure 5:**
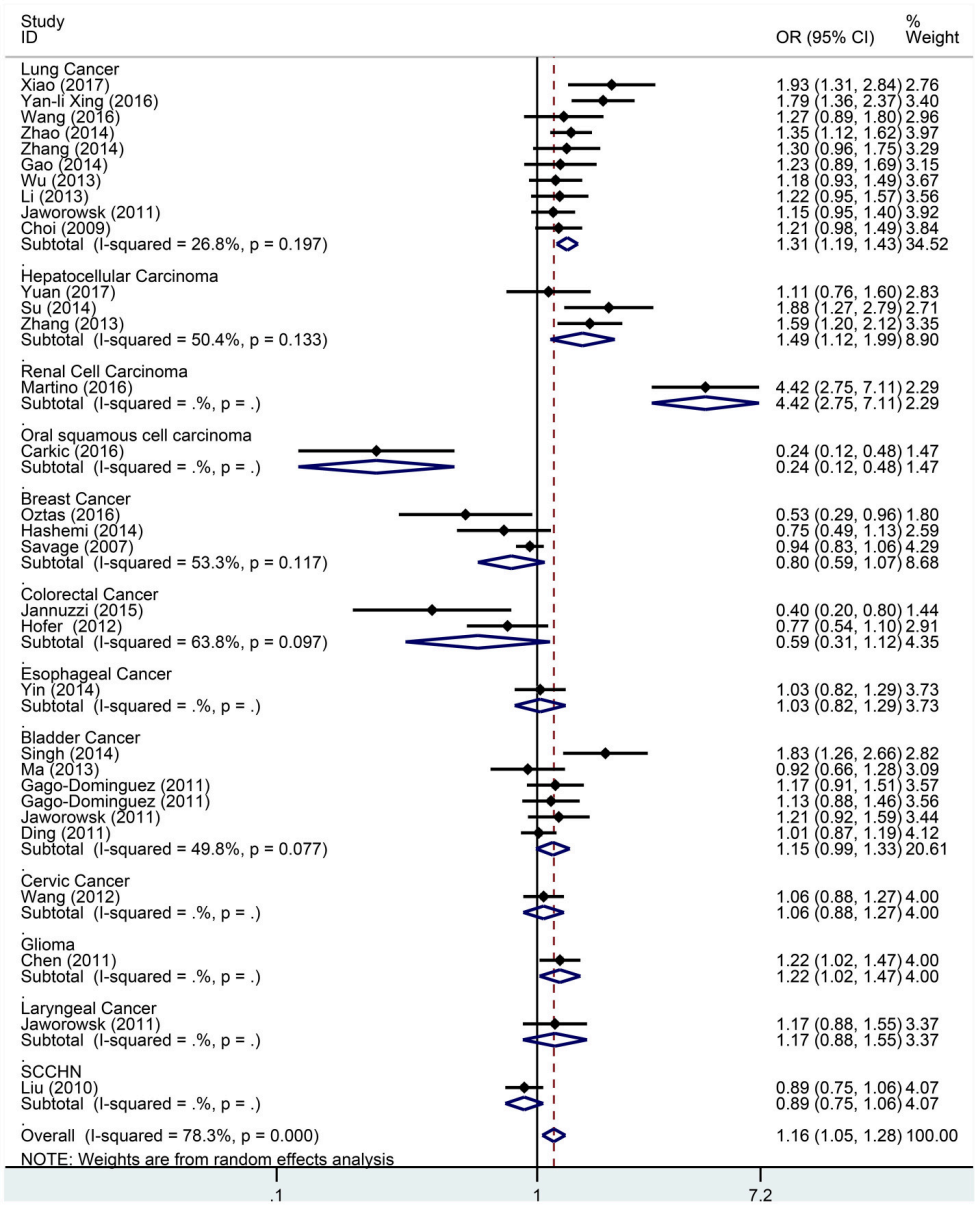
Forest plot for association between the TERT variant rs2736098 polymorphisms and cancer risk under a dominant genetic model (GA/AA vs. GG) after stratification analysis by cancer.

**Figure 6:**
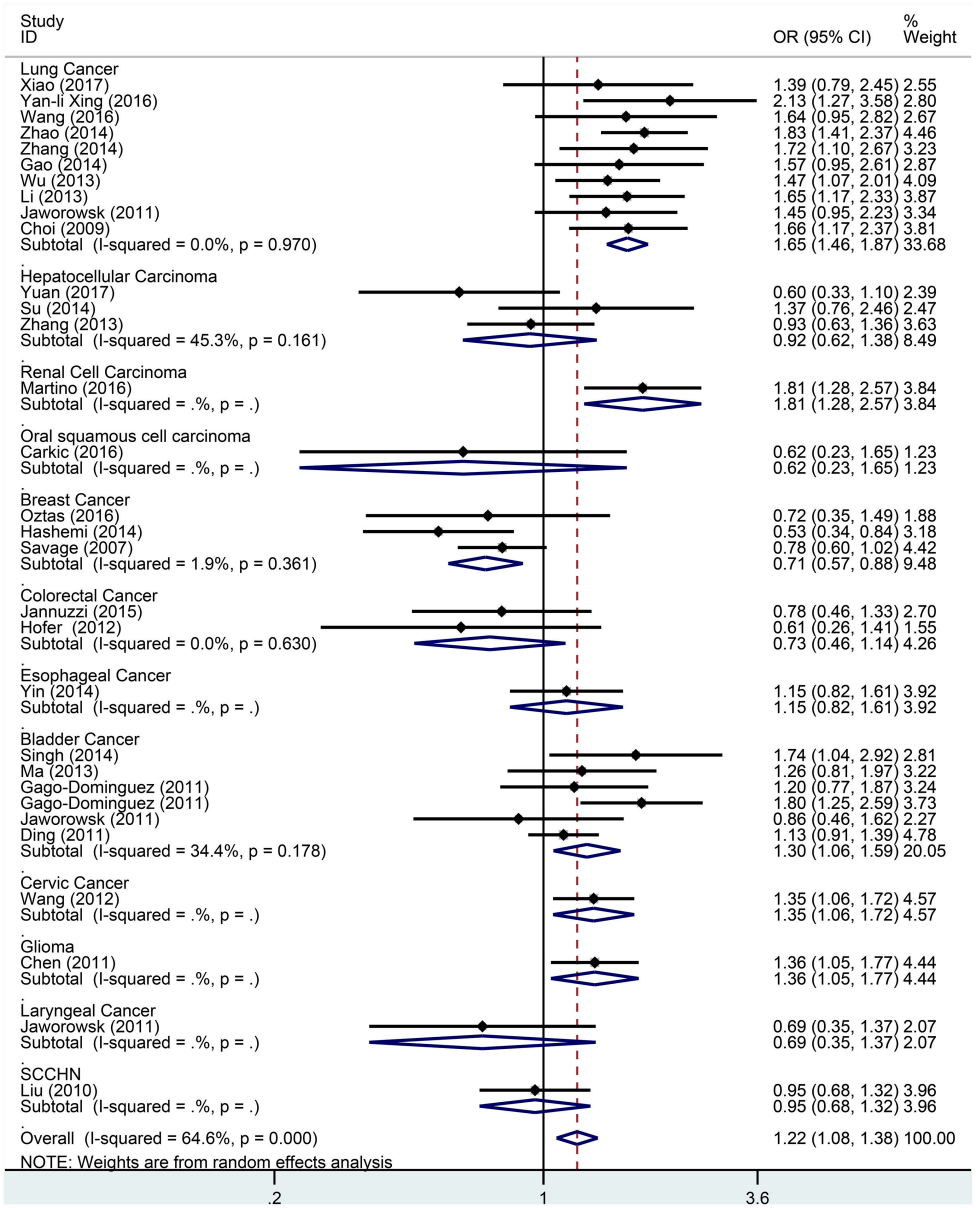
Forest plot for association between the TERT variant rs2736098 polymorphisms and cancer risk under a recessive genetic model (AA vs. GA/GG) after stratification analysis by cancer.

Subgroup analysis based on cancer type indicated that the TERT rs2736098 (G > A) polymorphism was associated with increased susceptibility to lung cancer under the allelic A vs. G genetic model (OR = 1.299, 95% CI = 1.216–1.386, *P* = 0.000), homozygous AA vs. GG genetic model (OR = 1.796, 95% CI = 1.575–2.047, *P* = 0.000), heterozygous GA vs. GG genetic model (OR = 1.199, 95% CI = 1.086–1.323, *P* = 0.000), dominant GA/AA vs. GG genetic model (OR = 1.305, 95% CI = 1.188–1.434, *P* = 0.000), and recessive AA vs. GA/GG genetic model (OR = 1.653, 95% CI = 1.461–1.869, *P* = 0.000). Similar results were obtained in bladder cancer under the allelic A vs. G genetic model (OR = 1.152, 95% CI = 1.032–1.286, *P* = 0.012), homozygous AA vs. GG genetic model (OR = 1.345, 95% CI = 1.058–1.708, *P* = 0.015), and recessive AA vs. GA/GG genetic model (OR = 1.297, 95% CI = 1.062–1.586, *P* = 0.011). In hepatocellular carcinoma, the heterozygous and dominant genetic models supported an increased cancer susceptibility in the population carrying the rs2736098 A allele (GA vs. GG: OR = 1.595, 95% CI = 1.246–2.042, *P* = 0.000; and GA/AA vs. GG: OR = 1.494, 95% CI = 1.123–1.987, *P* = 0.006). In colorectal cancer, the rs2736098 locus carrying the A allele was inversely related to colorectal cancer susceptibility in the allelic and homozygous models (A vs. G: OR = 0.717, 95% CI = 0.562–0.915, *P* = 0.007; and AA vs. GG: OR = 0.477, 95% CI = 0.276–0.825, *P* = 0.008). In breast cancer, the A allele of rs2736098 in the TERT gene showed protective effects against breast cancer in the allelic, homozygous, and recessive models (A vs. G: OR = 0.816, 95% CI = 0.670–0.994, *P* = 0.044; AA vs. GG: OR = 0.639, 95% CI = 0.458–0.892, *P* = 0.008; and AA vs. GA/GG: OR = 0.707, 95% CI = 0.566–0.884, *P* = 0.002). (Table 1, Figures 2–6).

In other cancer types including renal cell carcinoma, oral squamous cell carcinoma, esophageal cancer, glioma, cervical cancer, laryngeal cancer, and squamous cell carcinoma of the head and neck (SCCHN), a pooled analysis could not be performed because of the limited number of studies.

### Sensitivity analysis and publication bias

**Figure 7:**
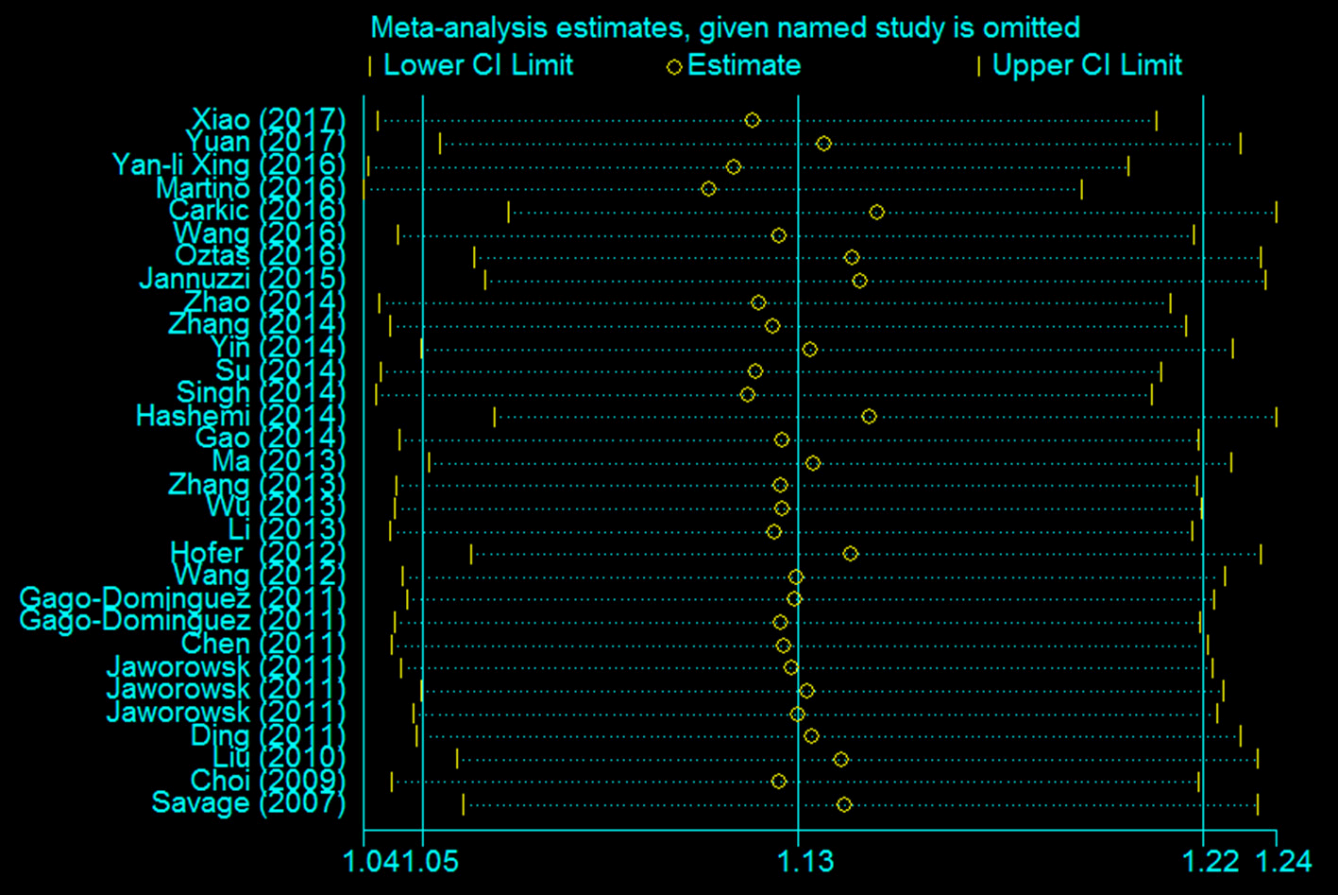
The sensitivity analysis for studies of the association between the TERT variant rs2736098 polymorphisms and cancer risk under a allele genetic model (A vs. G).

**Figure 8:**
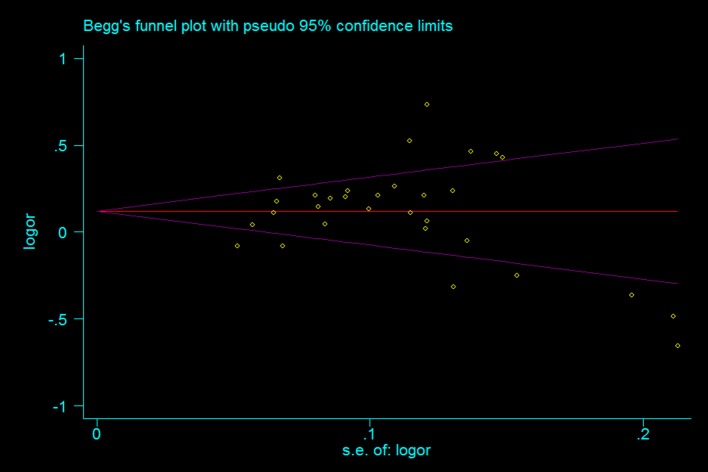
The Begg's funnel plot for studies of the association between the TERT variant rs2736098 polymorphisms and cancer risk under a allele genetic model (A vs. G). The horizontal and vertical axis represent the LogOR and confidence limits. OR, odds ratio; SE, standard error.

A leave-one-out analysis was performed to assess the effect of each individual study on the pooled ORs (Figure 7). The analysis results showed no significant alteration in the pooled ORs, indicating the statistical robustness of our results. Evaluation of publication bias showed no obvious publication bias in the funnel plot under the allelic genetic model (Figure 8).

## Discussion

TERT and CLTPM1L are the two promising candidate genes associated with cancer susceptibility in Chr5p15.33 (Wang et al., 2014). TERT encodes the catalytic protein subunit of telomerase and adds nucleotide repeats to chromosome ends in cooperation with a telomere RNA component (Cheung and Deng, 2008; Bao et al., 2017). TERT is commonly overexpressed in cancer cells, indicating genomic instability caused by telomerase activation, and telomere length change is associated with cancer risk and prognosis (Shay and Bacchetti, 1997; Wu et al., 2003). Because of its close association with telomere length, single nucleotide polymorphisms (SNPs) in TERT have been suggested as potential target genes for cancer therapy (Nan et al., 2011; Melin et al., 2012).

The rs2736098 (G > A) polymorphism, located in the second exon of TERT, is one of the most commonly investigated SNPs in the TERT gene, and its association with the risk of cancer was reported in various malignancies (Rafnar et al., 2009). Although rs2736098 polymorphism leads to synonymous mutation or silent mutation, without amino acid changing(Asn305Asn),this SNP may influence telomerase activity and shorten telemere length because of the location within the gene regulatory elements and alteration of transcription factor binding (Zhang et al., 2014; Wang et al., 2017). Evidence from two published meta-analyses indicated that the association between the variant rs2736098 and cancer susceptibility is inconsistent (Qi et al., 2012; Li et al., 2016). An increase in the number of studies on this association led us to establish strict inclusion and exclusion criteria to perform a comprehensive analysis and draw a relatively persuasive conclusion by avoiding the bias from inadequate studies.

Overall, this meta-analysis suggested a positive effect of rs2736098 genetic polymorphisms on overall cancer risk in five genetic models. However, this conclusion was challenged by the high heterogeneity among studies. Stratified analysis by ethnicity supported the strong correlation of TERT rs2736098 with cancer risk in the Asian population with acceptable heterogeneity, whereas this association was not found in the Caucasian population.

Subgroup analysis based on cancer type also mainly demonstrated cancer-enhancing effects in different cancer types. In lung cancer, this meta-analysis indicated that the TERT rs2736098 (G > A) polymorphism increased cancer susceptibility in the five genetic models. Xing et al. showed that carriers of the A allele of rs2736098 in the TERT polymorphism were more susceptible to non-small cell lung cancer (NSCLC) than carriers of the GG genotype under the dominant genetic model. Moreover, a potential gene-gene interaction between the A allele of rs2736098 and the G allele of rs2736100 contributed to an increased risk of NSCLC (Xing et al., 2016). One hospital-based study including 980 Chinese cases and 1,000 cancer-free cases showed that the homozygous TERT rs2736098 AA genotype was associated with an elevated susceptibility to lung cancer both in smokers and non-smokers (Zhao et al., 2014).

In bladder cancer, the A allele of rs2736098 in the TERT gene was 1.152 times more frequent than the G allele (OR = 1.152, 95% CI = 1.032–1.286), and similar results were obtained in the homozygous and recessive genetic models. Generally, the results from the current meta-analysis are consistent with previous studies performed in other populations. Singh et al. showed that the heterozygous genotype (GA) and the variant genotype (AA) in the TERT (G > A) polymorphism were significantly associated with a high risk for bladder cancer in the North Indian population (Singh et al., 2014).

With regard to hepatocellular carcinoma, the rs2736098 AA genotype was identified as an independent hereditary factor associated with carcinoma susceptibility in the Chinese population by Zhang et al. (2013) and Su et al. (2014). However, another study failed to demonstrate such genetic susceptibility, reporting only a significant association between the rs2736098 GA genotype and TERT promoter mutation-positive tumors compared with the AA genotype (Yuan et al., 2017). The current meta-analysis revealed that the TERT rs2736098 AA genotype was significantly associated with an increased risk of hepatocellular carcinoma in the heterozygous and dominant models.

However, inverse associations were observed in colorectal cancer and breast cancer. Ethnicity and sample size may explain the paradoxical effects of the rs2736098 polymorphism. In addition to lung cancer, bladder cancer, hepatocellular carcinoma, colorectal cancer, and breast cancer, the present meta-analysis also showed the association between the TERT rs2736098 polymorphism and the risk of renal cell carcinoma, oral squamous cell carcinoma, esophageal cancer, cervical cancer, glioma, laryngeal cancer, and SCCHN. However, pooled analysis could not be performed because of the limited number of cases of these cancers.

The present study had several limitations. Because a meta-analysis is a secondary analysis of original studies, the quality of the studies determines the credibility of this article. Although the original studies included had relatively large sample sizes, heterogeneity among studies cannot be avoided because of the variety in ethnicity, age distribution, genotyping methods, cancer types, and family history. Despite the fact that we performed subgroup analysis based on ethnicity and cancer type, other heterogeneity factors such as family history and genotyping methods may have affected the results. Furthermore, the occurrence of cancer is a comprehensive process resulting from multiple factors, and the effects of environmental factors on carcinogenesis could not be evaluated because of the lack of environment-related data from the included studies.

In conclusion, the results of this meta-analysis indicated significant associations between the TERT variant rs2736098 polymorphisms and cancer risk. The A allele in the rs2736098 G > A polymorphism contributed to cancer susceptibility in many malignancies, especially in lung and bladder cancers.

## Author contributions

XZ: Conceived and designed the study; MZ and BJ: Eligible study collection, and data extraction; XZ and MX: Statistical analyses; XZ, BJ, and MX: Preparation of tables and figures; XZ and MZ: Wrote and revised the manuscript. All authors reviewed the final manuscript.

### Conflict of interest statement

The authors declare that the research was conducted in the absence of any commercial or financial relationships that could be construed as a potential conflict of interest.
