# Supplementary material for: Association Between TERT rs2736098 Polymorphisms and Cancer Risk-A Meta-Analysis
Source: Front Physiol. 2018 Apr 11;9:377. doi: 10.3389/fphys.2018.00377 (PMC5905241; doi:10.3389/fphys.2018.00377)
Supplement: Supplementary file 1 [file DataSheet1.PDF]

Supplementary Material

An update meta-analysis of associations between the TERT variant rs2736098 polymorphisms and cancer risk

Mi Zhou1, Bo Jiang2, Mao Xiong2, Xin Zhu2*

1Department of Respiratory Medicine, the First Affiliated Hospital of Chongqing Medical University, Chongqing, China.

2Department of Urology, the First Affiliated Hospital of Chongqing Medical University, Chongqing, China.

*** Correspondence: Xin Zhu zhuxinxueyiwuhui@126.com**

5.1 Supplementary Figures

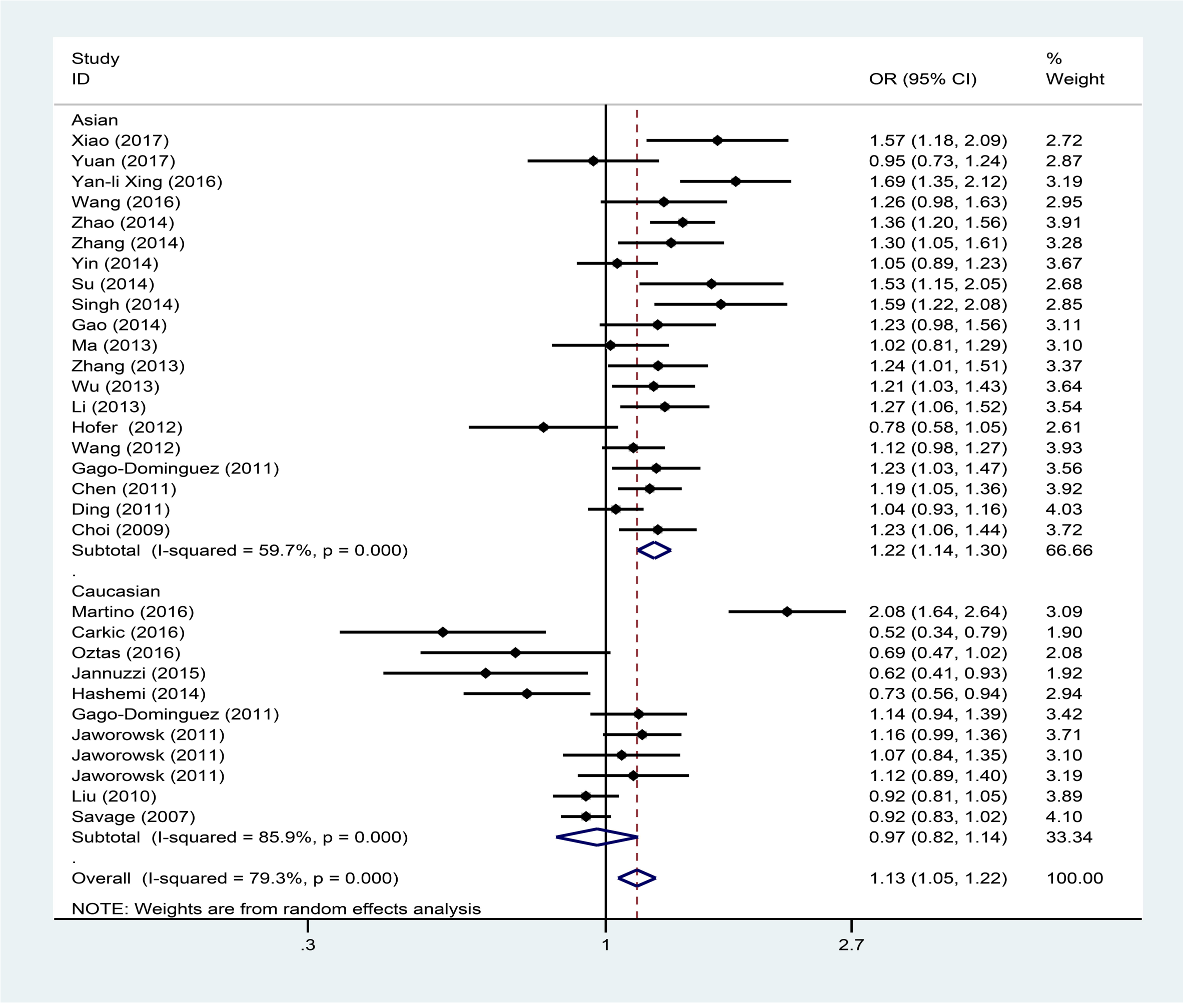

Supplementary FIGURE 1. Forest plot for association between the TERT variant rs2736098 polymorphisms and cancer risk under a allele genetic model (A vs. G) after stratification analysis by ethnicity.

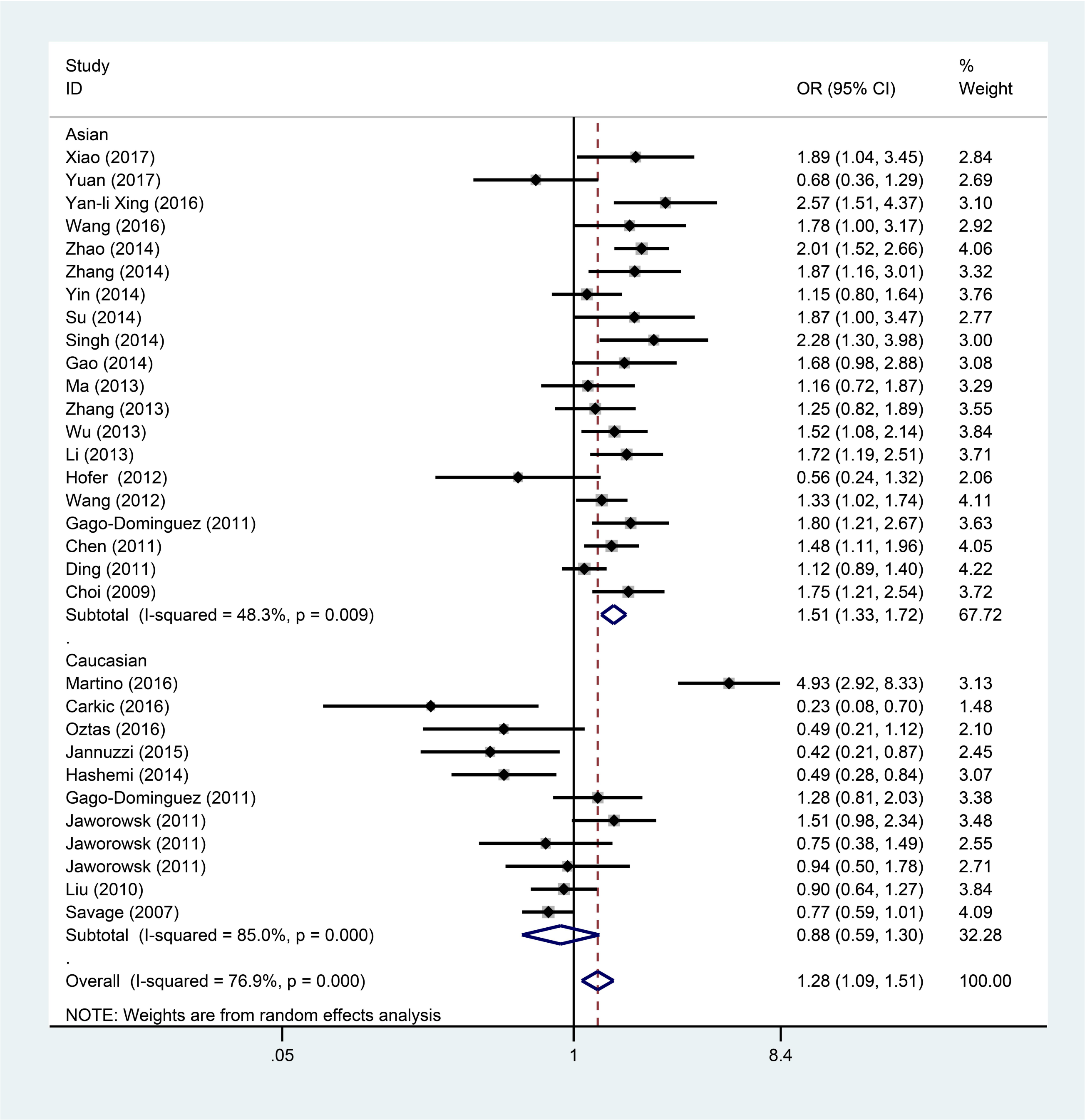

Supplementary FIGURE 2 . Forest plot for association between the TERT variant rs2736098 polymorphisms and cancer risk under a homozygote genetic model (AA vs. GG) after stratification analysis by ethnicity.

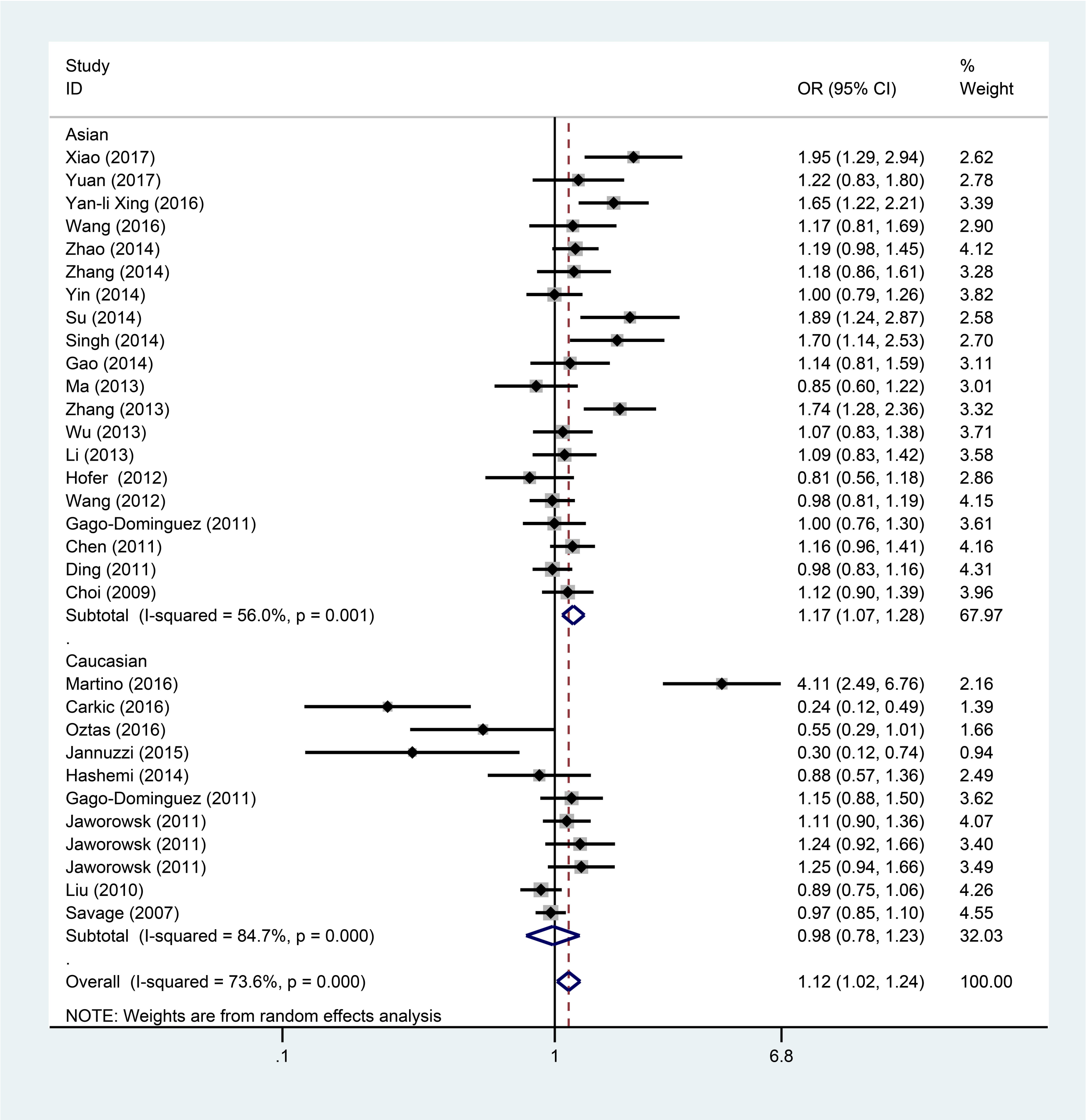

Supplementary FIGURE 3. Forest plot for association between the TERT variant rs2736098 polymorphisms and cancer risk under a heterozygote genetic model (GA vs. GG) after stratification analysis by ethnicity.

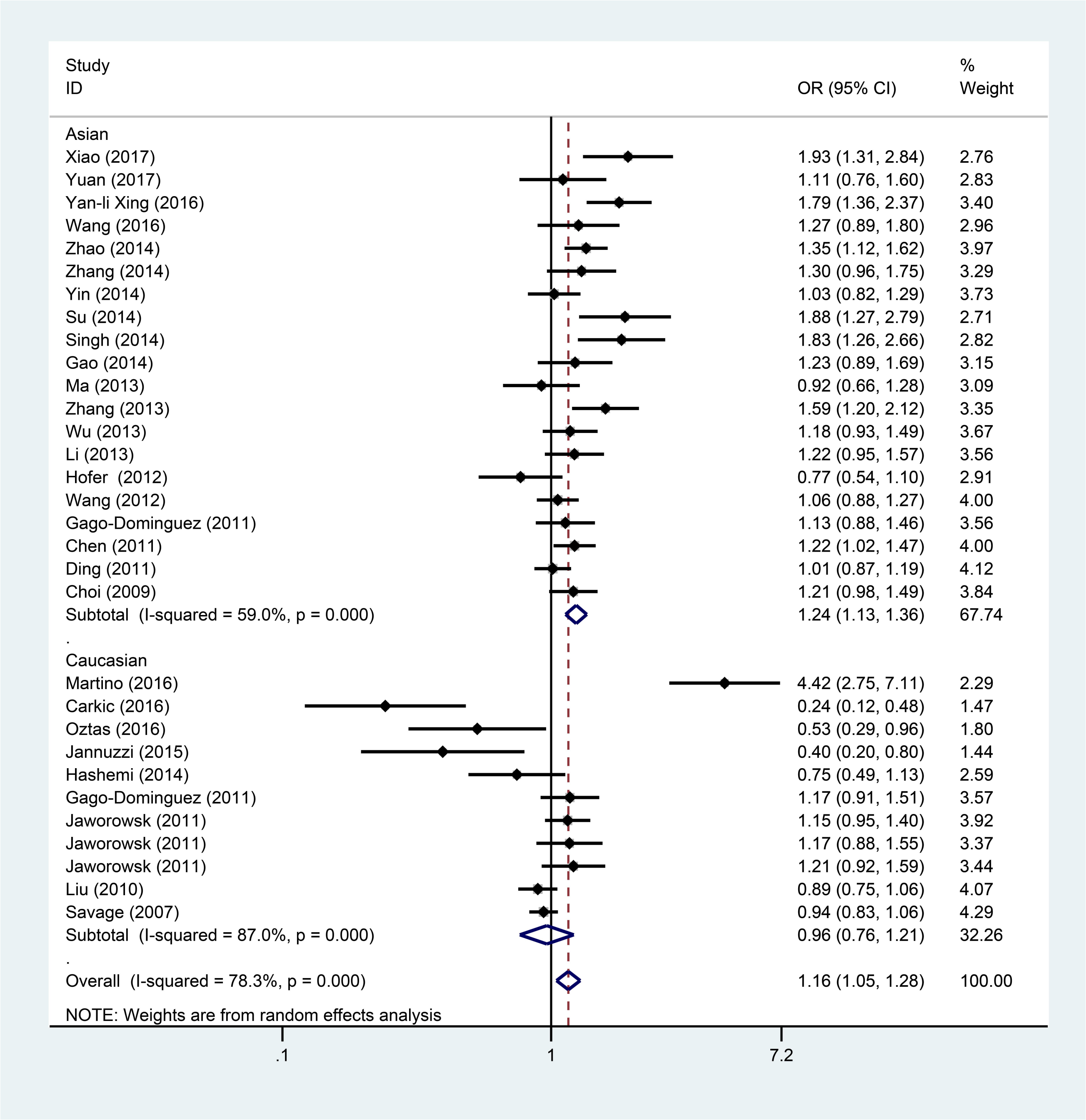

Supplementary FIGURE 4. Forest plot for association between the TERT variant rs2736098 polymorphisms and cancer risk under a dominant genetic model (GA/AA vs. GG) after stratification analysis by ethnicity.

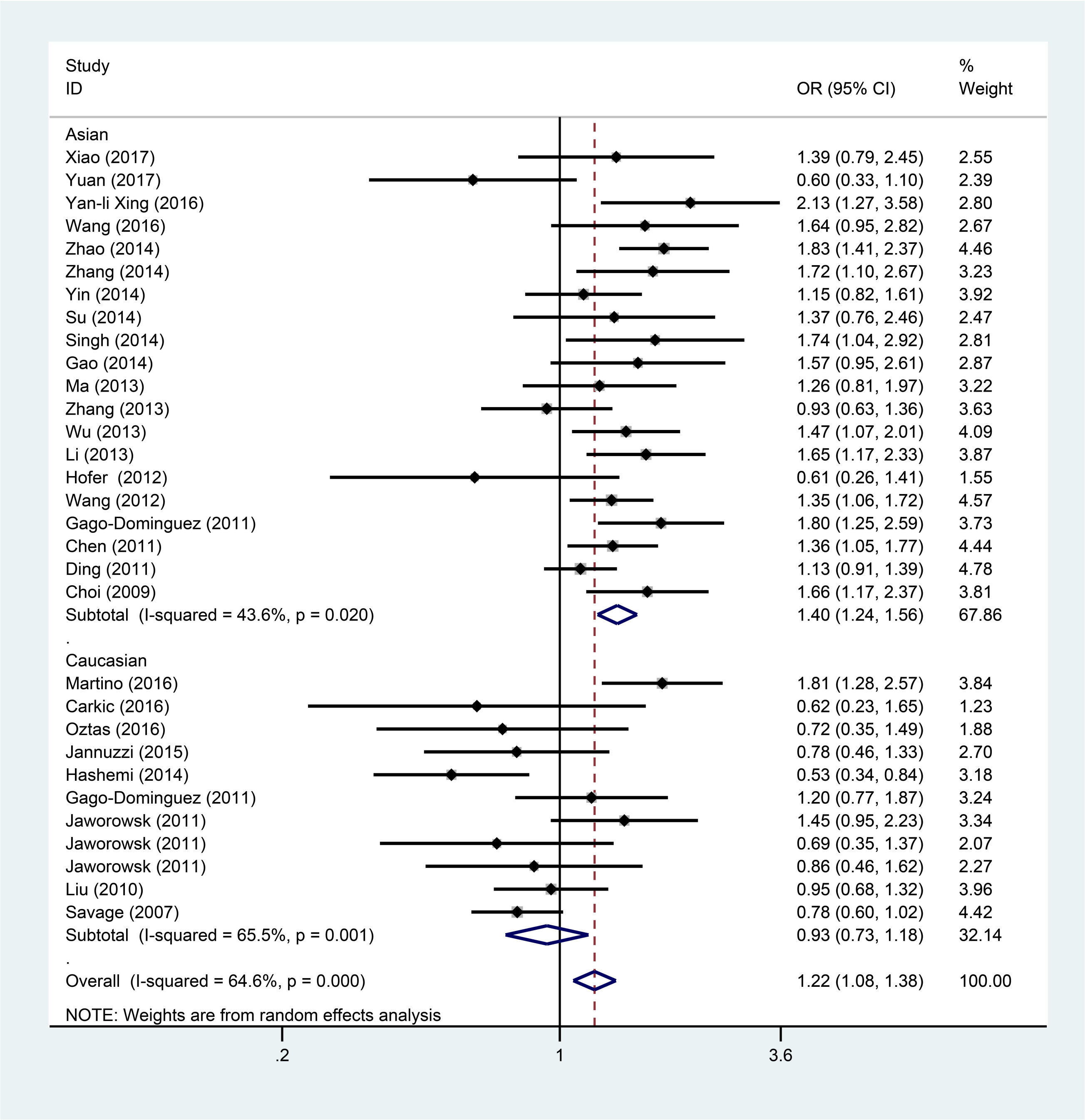

Supplementary FIGURE 5. Forest plot for association between the TERT variant rs2736098 polymorphisms and cancer risk under a recessive genetic model (AA vs. GA/GG) after stratification analysis by ethnicity.

Supplementary table 1| Characteristics of the investigated studies of the association between the TERT variant rs2736098 polymorphisms and cancer risk

First author	Year	Country	Ethnicity	Genotyping method	cancer type	Sample Size		Genotypes (Case/Control)						HWE (P)
						Case	Control	Case			Control			
								GG	GA	AA	GG	GA	AA	
Xiao	2017	China	Asian	TaqMan PCR	Lung cancer	203	225	78	95	30	123	77	25	0.02
Yuan	2017	China	Asian	TaqMan PCR	Hepatocellular Carcinoma	231	240	85	127	19	94	115	31	0.65
Yan-li Xing	2016	China	Asian	TaqMan PCR	Lung cancer	418	410	210	161	47	264	123	23	0.092
Martino	2016	Austria	Caucasian	TaqMan PCR	Renal Cell Carcinoma	239	366	24	123	92	121	151	94	0.001
Carkic	2016	Serbia	Caucasian	PCR-RFLP	Oral squamous cell carcinoma	90	100	38	45	7	15	73	12	<0.001
Wang	2016	China	Asian	Sequenom	Lung cancer	228	298	88	108	32	132	139	27	0.261
Oztas	2016	Turkey	Caucasian	PCR-RFLP	Breast cancer	107	108	40	52	15	26	62	20	0.115
Jannuzzi	2015	Turkey	Caucasian	PCR-RFLP	Colorectal cancer	104	135	25	14	65	15	28	92	<0.001
Zhao	2014	China	Asian	TaqMan PCR	Lung cancer	952	955	337	438	177	406	443	106	0.365
Zhang	2014	China	Asian	TaqMan PCR	Lung cancer	366	364	135	173	58	157	171	36	0.283
Yin	2014	China	Asian	LDR	Esophageal Cancer	600	651	245	277	78	270	306	75	0.403
Su	2014	China	Asian	TaqMan PCR	Hepatocellular Carcinoma	201	210	75	97	29	111	76	23	0.077
Singh	2014	India	Asian	TaqMan PCR	Bladder cancer	225	240	77	106	42	117	95	28	0.203
Hashemi	2014	Iran	Caucasian	PCR-RFLP	Breast cancer	252	222	72	140	40	51	113	58	0.777
Gao	2014	China	Asian	Sequenom	Lung cancer	309	308	122	145	42	137	143	28	0.28
Ma	2013	China	Asian	Sequenom	Bladder cancer	174	961	71	75	28	373	461	127	0.408
Zhang	2013	China	Asian	PCR-RFLP	Hepatocellular Carcinoma	400	400	133	206	61	177	158	65	0.004
Wu	2013	China	Asian	TaqMan PCR	Lung cancer	539	627	205	232	102	263	278	86	0.361
Li	2013	China	Asian	TaqMan PCR	Lung cancer	468	544	173	207	88	227	250	67	0.886
Hofer	2012	Austria	Asian	TaqMan PCR	Colorectal cancer	137	1705	86	45	6	963	623	119	<0.001
Wang	2012	China	Asian	TaqMan PCR	Cervic cancer	993	1015	375	444	174	397	480	138	0.71
Gago-Dominguez	2011	USA	Caucasian	TaqMan PCR	Bladder cancer	449	531	217	189	43	278	210	43	0.706
Gago-Dominguez	2011	China	Asian	TaqMan PCR	Bladder cancer	499	527	178	236	85	203	270	54	0.009
Chen	2011	China	Asian	Sequenom	Glioma	953	1033	351	461	141	430	486	117	0.246
Jaworowsk	2011	Poland	Caucasian	TaqMan PCR	Lung cancer	831	833	459	318	54	489	306	38	0.256
Jaworowsk	2011	Poland	Caucasian	TaqMan PCR	Laryngeal cancer	404	399	238	151	15	250	128	21	0.388
Jaworowsk	2011	Poland	Caucasian	TaqMan PCR	Bladder cancer	426	428	239	168	19	260	146	22	0.798
Ding	2011	China	Asian	TaqMan PCR	Hepatocellular Carcinoma	1273	1328	500	563	210	526	604	198	0.255
Liu	2010	Texas	Caucasian	TaqMan PCR	SCCHN	1079	1115	588	419	72	576	461	78	0.271
Choi	2009	Korea	Asian	PCR-RFLP	Lung cancer	720	720	311	322	87	345	320	55	0.102
Savage	2007	Poland	Caucasian	TaqMan PCR	Breast cancer	1967	2265	1171	699	97	1313	811	141	0.294

SCCHN= squamous cell carcinoma of the head and neck

Supplementary TABLE 1. Characteristics of the investigated studies of the association between the TERT variant rs2736098 polymorphisms and cancer risk.

TABLE 1 | Summary of meta-analysis of association between the TERT variant rs2736098 polymorphisms and cancer risk

Variables	N	A versus G			AA versus GG			GA versus GG			GA/AA versus GG			AA versus GA/GG		
		OR(95%Cl)	P_h	I^2(%)	OR(95%Cl)	P_h	I^2(%)	OR(95%Cl)	P_h	I^2(%)	OR(95%Cl)	P_h	I^2(%)	OR(95%Cl)	P_h	I^2(%)
All	31	1.134(1.051-1.224)	0	79.3	1.280(1.087-1.508)	0	76.9	1.125(1.020-1.240)	0	73.6	1.159(1.047-1.283)	0	78.3	1.223(1.082-1.384)	0	64.6
Cancer type																
Lung Cancer	10	1.299(1.216-1.386)	0.285	17.1	1.796(1.575-2.047)	0.907	0	1.199(1.086-1.323)	0.211	25.2	1.305(1.188-1.434)	0.197	26.8	1.653(1.461-1.869)	0.97	0
Bladder Cancer	6	1.152(1.032-1.286)	0.079	49.3	1.345(1.058-1.708)	0.096	46.5	1.097(0.940-1.279)	0.092	47.2	1.149(0.990-1.334)	0.077	49.8	1.297(1.062-1.586)	0.178	34.4
Breast Cancer	3	0.816(0.670-0.994)	0.107	55.2	0.639(0.458-0.892)	0.235	30.9	0.872(0.675-1.127)	0.194	39	0.796(0.592-1.070)	0.117	53.3	0.707(0.566-0.884)	0.361	1.9
Hepatocellular Carcinoma	3	1.211(0.948-1.548)	0.057	65.1	1.179(0.711-1.956)	0.081	60.2	1.595(1.246-2.042)	0.254	27.1	1.494(1.123-1.987)	0.133	50.4	0.921(0.617-1.376)	0.161	45.3
Colorectal Cancer	2	0.717(0.562-0.915)	0.371	0	0.477(0.276-0.825)	0.611	0	0.539(0.207-1.401)	0.047	74.6	0.592(0.312-1.121)	0.097	63.8	0.726(0.462-1.141)	0.63	0
Renal Cell Carcinoma	1	2.081(1.642-2.637)	-	-	4.934(2.922-8.332)	-	-	4.107(2.495-6.761)	-	-	4.424(2.752-7.112)	-	-	1.811(1.275-2.571)	-	-
Oral squamous cell carcinoma	1	0.518(0.341-0.786)	-	-	0.230(0.076-0.697)	-	-	0.243(0.120-0.492)	-	-	0.241(0.121-0.482)	-	-	0.618(0.232-1.647)	-	-
Esophageal Cancer	1	1.047(0.889-1.234)	-	-	1.146(0.799-1.645)	-	-	0.998(0.787-1.265)	-	-	1.027(0.820-1.286)	-	-	1.148(0.818-1.610)	-	-
Cervic Cancer	1	1.118(0.984-1.269)	-	-	1.335(1.025-1.739)	-	-	0.979(0.809-1.186)	-	-	1.059(0.884-1.267)	-	-	1.350(1.059-1.721)	-	-
Glioma	1	1.194(1.050-1.359)	-	-	1.476(1.113-1.959)	-	-	1.162(0.961-1.405)	-	-	1.223(1.021-1.465)	-	-	1.359(1.045-1.768)	-	-
Laryngeal Cancer	1	1.066(0.842-1.351)	-	-	0.750(0/378-1.490)		-	1.239(0.923-1.664)	-	-	1.170(0.881-1.554)	-	-	0.694(0.353-1.367)	-	-
SCCHN	1	0.923(0.807-1.055)	-	-	0.904(0.643-1.271)	-	-	0.890(0.747-1.061)	-	-	0.892(0.755-1.055)	-	-	0.951(0.682-1.325)	-	-
Ethnicity																
Asian	20	1.217(1.138-1.301)	0	59.7	1.509(1.327-1.716)	0.009	48.3	1.170(1.066-1.284)	0.001	56	1.240(1.132-1.358)	0	59	1.396(1.245-1.565)	0.02	43.6
Caucasian	11	0.965(0.818-1.140)	0	85.9	0.875(0.588-1.302）	0	85	0.984(0.785-1.235)	0	84.7	0.964(0.765-1.214)	0	87	0.928(0.730-1.181)	0.001	65.5

CI=confidence interval, OR=odds ratio, I^2=the variation in OR attributable to heterogeneity   SCCHN= squamous cell carcinoma of the head and neck

TABLE 1. Summary of meta-analysis of association between the TERT variant rs2736098 polymorphisms and cancer risk.
